# The Role of Novel Cardiac Imaging for Contemporary Management of Heart Failure

**DOI:** 10.3390/jcm11206201

**Published:** 2022-10-20

**Authors:** Frank A. Flachskampf, Tomasz Baron

**Affiliations:** 1 Department of Medical Sciences, Cardiology and Clinical Physiology, Uppsala University Hospital, Uppsala University, 751 85 Uppsala, Sweden; 2 Uppsala Clinical Research Center, Uppsala University, 752 36 Uppsala, Sweden

**Keywords:** heart failure, cardiac imaging, echocardiography, cardiac magnetic resonance, computed tomography, positron emission tomography

## Abstract

Heart failure is becoming the central problem in cardiology. Its recognition, differential diagnosis, and the monitoring of therapy are intimately coupled with cardiac imaging. Cardiac imaging has witnessed an explosive growth and differentiation, with echocardiography continuing as the first diagnostic step; the echocardiographic exam itself has become considerably more complex than in the last century, with the assessment of diastolic left ventricular function and strain imaging contributing important information, especially in heart failure. Very often, however, echocardiography can only describe the fact of functional impairment and morphologic remodeling, whereas further clarification of the underlying disease, such as cardiomyopathy, myocarditis, storage diseases, sarcoidosis, and others, remains elusive. Here, cardiovascular magnetic resonance and perfusion imaging should be used judiciously to arrive as often as possible at a clear diagnosis which ideally enables specific therapy.

## 1. Introduction

Heart failure, typically and somewhat vaguely defined as a condition in which the heart is unable to pump enough blood to meet the body’s needs, has grown into one of the most important cardiovascular health challenges, in part due to an ageing population, in part due to the improved treatment of conditions formerly leading to earlier death, such as myocardial infarction, which now are more likely to being survived, but nevertheless may lead to later heart failure. In Sweden, about 2% of the population are affected by clinically manifested heart failure [1].

“Heart failure” comprises a multitude of etiologies and pathophysiologies, and is in itself a rather broad concept defined by a constellation of clinical symptoms and signs. Therefore, to provide management and therapy tailored to individual etiology, as specific a diagnosis as possible is necessary. In the majority of cases, cardiac imaging is necessary—and often sufficient—to arrive at such specific diagnoses, complemented by laboratory chemistry and invasive diagnostics, where necessary. Cardiac imaging in heart failure has evolved over roughly the last 50 years from chest X-ray, until very recently a nearly always-obtained test, to the impressive armamentarium available—in spite of some restrictions—today. In this article, we will review the capabilities of current cardiac imaging according to clinical questions in the context of heart failure.

## 2. Is It Heart Failure?

Remarkably, the basic diagnosis of heart failure remains largely a clinical one based on history, physical examination, and natriuretic peptides. Although, for example, pulmonary congestion is very well detectable by radiology or lung ultrasound, milder forms of heart failure are difficult to pin down with confidence without suggestive symptoms and signs. In this context, natriuretic peptides have largely superseded the use of the chest X-ray to screen for heart failure, although they are multifactorial, sex- and body-mass-dependent, may increase in response to heart failure medication (e.g., beta blockers), have a large scatter, and have limited accuracy in milder forms of heart failure [2].

## 3. Valvular Heart Disease

Severe—and sometimes moderate—primary valvular heart disease is an important cause or contributor to heart failure. Since this etiology is frequently and well described in the current literature, this review does not include a detailed discussion of valvular heart disease.

## 4. Left and Right Ventricular Function

Left ventricular (LV) “function” is a fuzzy term, but overwhelmingly important in cardiologists’ thinking. Systolic LV function is mostly equated with ejection fraction (EF) or global longitudinal strain (GLS), although an argument could be made for using stroke volume or stroke work instead. Stroke volume, and its product with heart rate, cardiac output, is “what the body sees” of the ventricles’ performance, and is typically reduced in heart failure with reduced EF, but also in heart failure with preserved EF, thus offering a more uniform physiologic correlate of the clinical syndrome of heart failure. As a clinical illustration, take the scenarios of manifested cardiac amyloidosis or of papillary muscle rupture, both leading to normal or even high ejection fraction, together with critically reduced stroke volume. This can be further refined by correcting for afterload, e.g., by multiplying stroke volume by mean arterial blood pressure to yield stroke work.

“Diastolic function”, an even less clear term, describes the complex variations in LV diastolic pressure–volume relationships, and is typically assessed by indirect echocardiographic signs, which have modest accuracy at best [3,4].

Heart failure diagnosis is now tightly related to the determination of left ventricular ejection fraction (EF) by virtue of the current stratification of heart failure into three subgroups of heart failure according to their EF, heart failure with reduced EF (HFrEF), heart failure with mildly reduced EF (HFmrEF), and heart failure with preserved EF (HFpEF) [2].

Ejection fraction is routinely measured by 2D echocardiography, with acceptable accuracy compared to the gold standard CMR. LV volumes (both end-diastolic and end-systolic), however, are regularly and substantially underestimated by 2D echocardiography. This can be partly overcome by 3D echocardiography and/or left heart contrast use. 3D echocardiography also compensates for irregularities in ventricular shape, such as in aneurysms, but depends on good image quality.

Additionally, the newer speckle tracking-based deformation parameter, global longitudinal shortening (GLS), serves mainly as a sort of canary in the mine in the presence of borderline or slightly reduced EF; for example, in valvular heart disease or cardiotoxicity of cancer therapy, where GLS decreases earlier and by a higher proportion than EF in the early stages and, therefore, regularly pre-dates later EF reductions [5]. In direct comparison, the prognostic value of GLS regularly outperforms EF [6] in patients with heart failure, probably because of better discrimination at the lower limits of normalcy. The reasons for this superiority of GLS over EF are not entirely clear. Higher sensitivity of longitudinal LV function for subtle functional changes seems to be essential, since longitudinal function is more directly measured by GLS than by EF.

**Figure 1:**
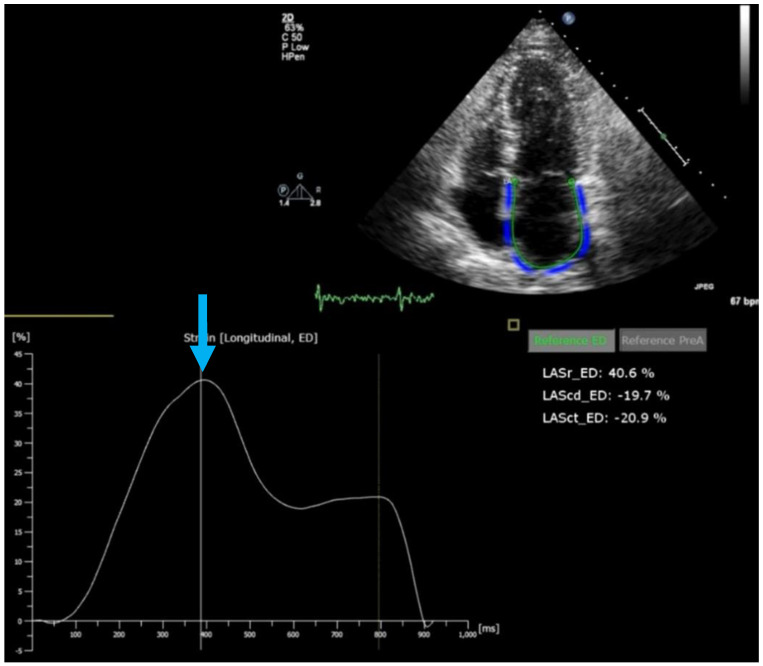
Left atrial strain by speckle tracking echocardiography. Upper panel: echocardiographic image (apical four-chamber view) showing the left ventricle (LV) and left atrium (LA) with blue coded region of interest for LA strain tracking. Lower panel: LA strain trace along with ECG. Reservoir strain (41%) is indicated by the blue arrow.

**Figure 2:**
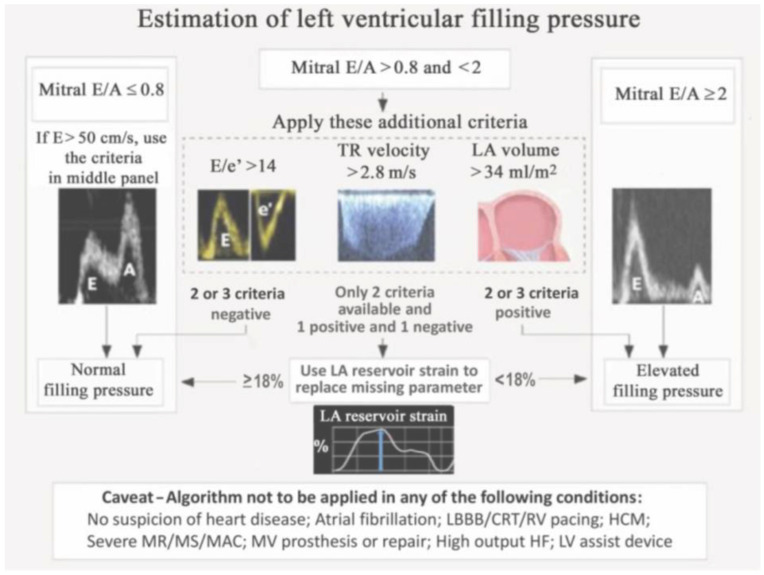
Current algorithm of the European Association of Cardiovascular Imaging for echocardiographic assessment of left ventricular filling pressure in patients with suspected heart failure with preserved ejection fraction. Note that this algorithm integrates left atrial reservoir strain, with a cut-off of 18%, as a reserve parameter if one of the classic parameters, LA volume, E/e’, or systolic pulmonary arterial pressure, cannot be obtained with confidence. Reproduced, with permission, from [8].

**Figure 3:**
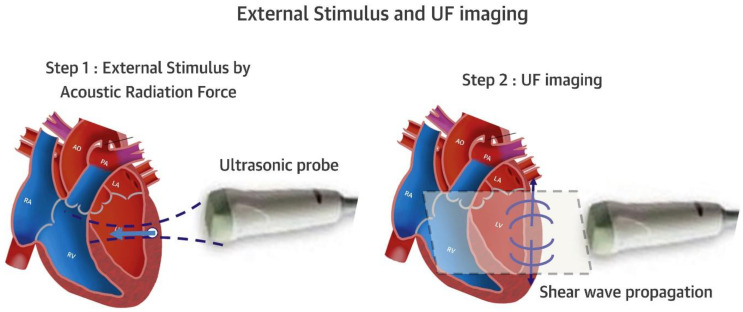
Schematic drawing of externally stimulated shear wave imaging. The shear wave is initiated by an external stimulus, and then visualized by very-high-framerate imaging. This allows imaging the propagation of the shear wave, and to obtain shear wave speed. From shear wave speed, under simplifying assumptions, the stiffness of a tissue can be calculated. UF ultrafast. Adapted with permission from [14].

The assessment of diastolic function has recently been improved, with added prognostic value, by adding left atrial (LA) strain to the diagnostic algorithm ([7,8]; Figure 1 and Figure 2). Other important developments in the non-invasive diagnosis of diastolic LV dysfunction are the use of provocative maneuvers such as physical exercise or volume challenge [9,10,11]. Such maneuvers aim to unmask diastolic dysfunction which may not be evident at rest by increasing cardiac workload. Under provocation by exercise or volume, LV stiffness increases and leads to measurable changes in indirect parameters such as E/e’ or right ventricular systolic pressure. Other experimental techniques assess the twisting and untwisting motion of the LV around its long axis, which are important for both systolic (twist) and diastolic (untwist) LV function; this can be accomplished by speckle-tracking echocardiography or CMR [12]. Finally, shear wave imaging, which harnesses the information on tissue stiffness from the velocity of pressure waves in the myocardium, may be useful if it can be standardized and technically integrated in ultrasound machines ([13,14]; Figure 3).

An important, but underappreciated, area of echocardiographic imaging is patients with impaired LV function with left bundle branch block who are candidates for cardiac resynchronization therapy. Although past trials have had mixed results for the predictability of CRT success from baseline echocardiographic parameters, several visual features (“mechanical abnormalities”) have been described which imply an increased likelihood for a benefit from CRT: (1) the septal flash, (2) “apical rocking”, etc. [15].

Right ventricular (RV) function is more difficult to quantify, given the more limited visualization by echo, the complex RV morphology with separate inflow and outflow tract, and the stronger impact of preload and afterload on all RV functional parameters. The classic echo parameters, such as tricuspid annular plane systolic excursion (TAPSE) and fractional area change, have been complemented by RV strain, both in its “global” (six segments) or free wall strain (three segments) form, but the obtained strain only reflects myocardial shortening of the RV seen in the apical four-chamber view, i.e., the inflow tract of the RV. Even more limited, and essentially a close relative of TAPSE, is the basal RV free wall’s systolic peak velocity S’. Three-dimensional echocardiography, although often impractical due to image quality, can provide the EF of the entire RV, which is generally accepted as the best parameter of RV function. RVEF can be obtained with greater accuracy and more reliable absolute RV volumes by CMR and CT. Therefore, if RV performance needs to be assessed and monitored with high precision, e.g., in postoperative Fallot tetralogy patients with substantial pulmonary regurgitation, CMR is now the preferred modality. It should be remembered, however, that all of the cited global left or right ventricular functional parameters are clearly dependent on preload and afterload, and have substantial test–retest variability [16,17]. Parameters which include or correct for afterload, such as stroke work or cardiac power output [18,19], or true myocardial external efficiency based on myocardial oxygen uptake [20] obtained by positron emission tomography (PET) have not reached clinical implementation, in spite of some encouraging data in the literature. Very recently, the myocardial work “index” derived from pressure-strain loops of the left ventricle, which can be obtained non-invasively by the extrapolation of cuff blood pressure recordings, has been proposed as a parameter incorporating blood pressure as a measure of afterload. This approach, however, was originally developed to detect regional differences in mechanical work, such as those occurring in left bundle branch block. It does not take into account absolute ventricular volumes, and, therefore, is best suited for intra-individual comparisons, e.g., before and after cardiac resynchronization, where “wasted work” of the septum in left bundle branch block conditions can be measured, and the benefit from resynchronization quantified regionally [21,22,23,24,25]. For the performance of the right ventricle, recently, the ratio of TAPSE to systolic pulmonary arterial pressure (SPAP), sometimes described as an index of ventriculo-arterial loading, has been proposed [26]. This index has shown prognostic value, e.g., in patients with heart failure [26], medically treated tricuspid regurgitation [27], and in patients undergoing mitral edge-to-edge repair for functional mitral regurgitation [28], but not in other studies, e.g., in the context of transcatheter tricuspid valve repair [29].

## 5. Cardiac Imaging for Monitoring Heart Failure

In some clinical situations, it is desirable to not only monitor patients’ symptoms (and natriuretic peptides) under heart failure therapy, but also to quantify objectively functional improvements and reverse remodeling of the heart. An example of this is the recommendation to defer implantation of a prophylactic implantable cardioverter/defibrillator in patients with newly diagnosed HFrEF with EF < 35% for at least 3 months to allow for recovery of LV function under “optimal medical therapy”. It should be pointed out that though therapy with angiotensin-converting enzyme inhibitors, beta blockers, and sacubitril/valsartan leads to a well-documented improvement in ejection fraction, this effect seems to be absent or minor in SGLT2 inhibitors [30,31].

## 6. Structure of the Myocardium

Though echocardiography delineates well the border between blood and tissue, and furnishes fundamental, if not very precise, information about linear dimensions and volumes of chambers, the characterization of the chamber walls themselves is very limited. In a very approximative way, LV mass is assessed using linear dimensions at the base of the LV, and LV hypertrophy is, thus, diagnosed clinically. Note, however, that this is essentially a misnomer, since we are simply converting wall volume into mass by multiplying cm^3^ (or mL) by 1.04 g/mL to arrive at grams. This volume-derived mass may be hypertrophied myocytes, as in hypertension or aortic stenosis, but it may also be due to an increase in extracellular space, as in cardiac amyloidosis, myocardial edema, diffuse or localized (“replacement”) fibrosis, intracellular glycosphingolipids in Fabry disease [32], and others. Echocardiography may provide clues about specific diseases; for example, in cardiac amyloidosis, it has been observed that apical LV segments tend to have preserved longitudinal strain, whereas mid and basal segments display reduced strain (“apical sparing”; [33]). However, this sign lacks specificity in hypertrophied ventricles, and, in amyloidosis, becomes positive mainly in advanced disease. In some cases, as in aortic stenosis, we know that, besides true myocyte hypertrophy, 10–15% of patients also have cardiac amyloidosis [34], and, thus, these patients have both a myocardial and extracellular increase in mass. This blind spot for echocardiography can be removed to an extent by CMR and perfusion imaging.

**Figure 4:**
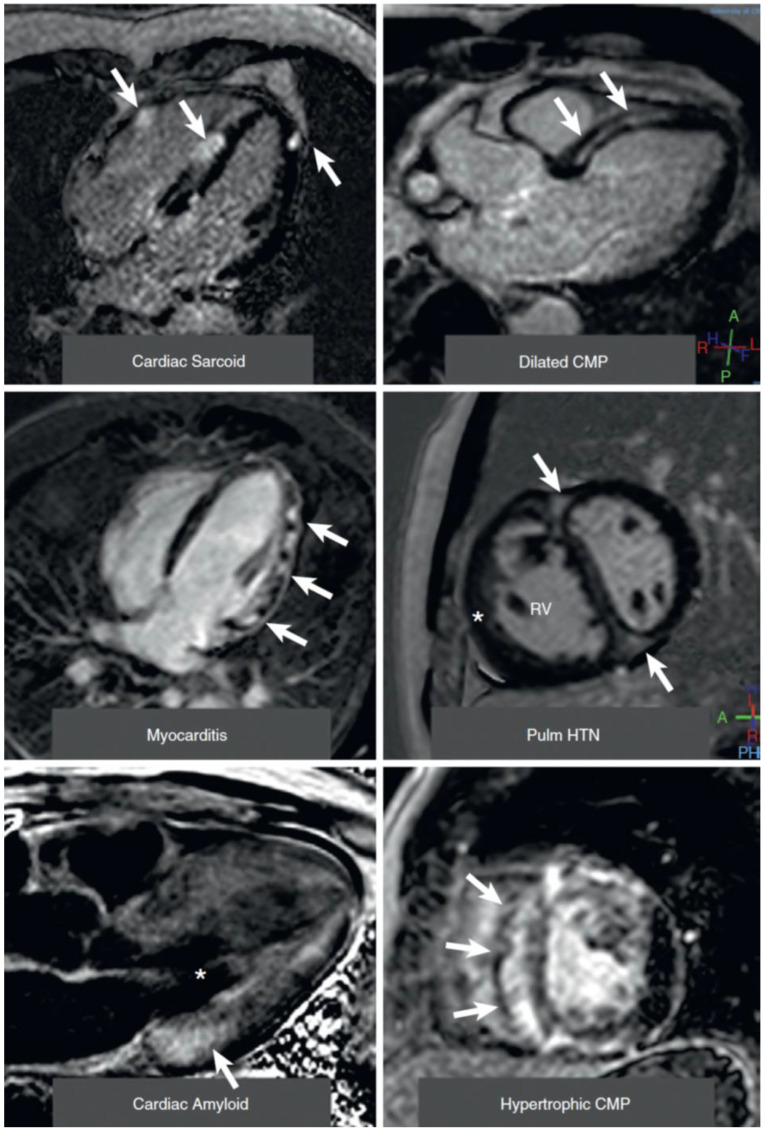
CMR images with late gadolinium enhancement (LGE) in non-ischemic cardiomyopathies. Top left: cardiac sarcoidosis, with patchy distribution of LGE (arrows) in lateral wall and septum. Top right: septal mid-wall “stripe” pattern of LGE (arrows) in dilated cardiomyopathy. Middle left: patchy epicardial and mid-wall LGE (arrows) in the lateral wall in myocarditis. Middle right: midventricular short-axis view in a patient with pulmonary hypertension, right ventricular dilatation, and hypertrophy (star). Note LGE (arrows) at the right ventricular insertion points. Bottom left: cardiac amyloid. The LV blood pool is nulled (star) and there is subtle circumferential subendocardial LGE throughout the LV, most pronounced at the base (arrow). Bottom right: midventricular short-axis view of patient with hypertrophic cardiomyopathy and septal hypertrophy showing extensive mid-wall LGE (arrows). Reproduced, with permission, from [38].

**Figure 5:**
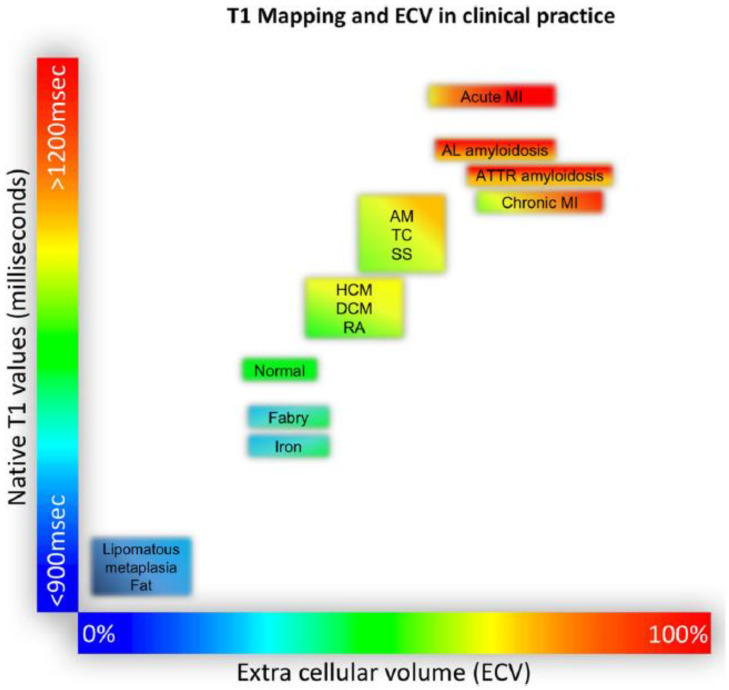
Tissue characterization using native T1 and extracellular volume fraction (ECV). For the purpose of comparability, only studies using 1.5 T scanners were considered in this figure. AM, acute myocarditis; DCM, dilated cardiomyopathy; HCM, hypertrophic cardiomyopathy; MI, myocardial infarction; RA, rheumatoid arthritis; TC, takotsubo cardiomyopathy; SS, systemic sclerosis. Reproduced according to the Creative Commons CC BY license from [39].

“Virtual histology” by CMR is based on two principles [35]:

1. Early and late tissue enhancement after contrast (gadolinium) application allows separation of intact myocardium from extracellular space, since intact myocardiocytes do not allow the intracellular accumulation of gadolinium. Thus, late gadolinium enhancement can detect myocardial scars as locally increased extracellular volume, such as infarcts, post-myocarditis damage, sarcoidosis, and other localized increases of extracellular volume (Figure 4). This technique relies on relative contrast intensity differences between myocardial regions, as in a myocardial infarction, but does not work in the presence of diffuse alterations of extracellular space. Though all of the cited etiologies result in the formation of localized increases of extracellular volume, often the “gestalt” of such a region of late contrast enhancement suggests a specific etiology. For example, subendocardial or transmural late enhancement following the “wavefront phenomenon” of myocardial ischemia in a coronary perfusion territory is strongly suggestive of post-infarct scar, and mid-wall late enhancement, especially in the lateral wall, suggests post-myocarditis damage. Note that similar principles enable contrast-enhanced CT to diagnose localized increases in extracellular space [36] in a similar manner to CMR, although the achieved contrast between normal and diseased regions is weaker than with CMR techniques.
2. Pixel-wise maps of the magnetic relaxation parameters T1, T2, and T2*, called “parametric imaging”, allow a limited characterization of underlying tissue ([37]; Figure 5). Additionally, by pre- and post-contrast registration of T1 in the myocardium and blood pool, a direct relative measure of extracellular volume (in percent) for each pixel can be calculated. For example, hemochromatosis leads to shortening and amyloidosis leads to lengthening of T1 relaxation times, which sets these pathologies apart from others. Myocardial edema leads to lengthening of T1 and T2 times and the expansion of extracellular volume, which in themselves are non-specific, but may aid in the diagnosis of, e.g., myocarditis, depending on clinical circumstances. Notably, increased extracellular space may be indicative of diffuse fibrosis but also, e.g., tissue edema. Hence, although, for example, an increased T1 value does correlate modestly with myocardial fibrosis and may, therefore, support the diagnosis of diastolic dysfunction and HFpEF, the relaxation parameters are multifactorial and should not be mistaken for true histology.

GLS by echocardiography or CMR has been shown to correlate with diffuse myocardial fibrosis, both for the left ventricle and for the left atrium [40,41], but the correlation is modest at best and is difficult to apply for individual clinical decisions. Similarly, clear signs of diastolic dysfunction, such as short E wave deceleration, high E/e’ ratio, low left atrial strain, or increased systolic right ventricular pressure, are suggestive of increased diastolic LV pressures, which, in turn, are indicative of myocardial fibrosis, if other causes can be excluded. It should be clear, though, that CMR relaxation times or echocardiographic GLS are not specific histologic markers, and are, at best, modestly related to histological fibrosis.

## 7. Myocardial Perfusion

Coronary artery disease (CAD) is an important cause of heart failure, and the classic way to non-invasively diagnose CAD is diagnosing inducible ischemia. This is traditionally performed either by looking at LV wall motion abnormalities during physical or pharmacological stress by echocardiography [42], or, less frequently, by gated single-photon emission tomography (SPECT), or CMR cine-loops. Left heart echo contrast application improves the diagnostic accuracy of stress echocardiography, and should be strongly considered even in patients with acceptable image quality at rest [43]. The injection of contrast, besides opacification of the chambers, leads to myocardial opacification, and this can be used to assess myocardial perfusion. The technique is highly dependent on experienced operators, however, since the destruction of microbubbles by the ultrasound itself may mimic hypoperfusion. Several other refinements have been proposed, most importantly the recording of coronary flow velocity in the mid-left anterior descending coronary artery, coupled with a vasodilator stress (adenosine or dipyridamole) to determine coronary flow velocity reserve. Again, a learning curve exists, and the method has not found widespread acceptance.

Alternatively, perfusion can be directly visualized by nuclear imaging (SPECT or PET) or contrast CMR. Using specialized tracers, PET allows to obtain estimates of absolute regional perfusion (in mL blood per second and gram myocardium), and, thus, is able to assess perfusion at the myocardial level, enabling the evaluation of the whole cardiac vasculature. Further, contrast CMR [44] has been shown not only to be a highly accurate diagnostic method to diagnose ischemia, but also to provide strong prognostic data, and reduce the number of unnecessary invasive coronary angiographies in patients with stable chest pain [45]. Moreover, recently, quantitative myocardial blood flow mapping using CMR has also been introduced, offering the ability to separate epicardial multivessel disease from microvascular disease besides prognostic stratification [46,47].

A specific strength of PET is the possibility to characterize regional perfusion and metabolism using different tracers, enabling the detection of a myocardium which is metabolically active, but underperfused and non-functional, the classic hallmark of a viable myocardium. Although the clinical utility of diagnosing myocardial viability has been questioned due to lack of data showing prognostic benefit, most prominently in the STICH trial [48], there is ongoing clinical interest [49] in this scenario.

## 8. Other Nuclear Imaging

**Figure 6:**
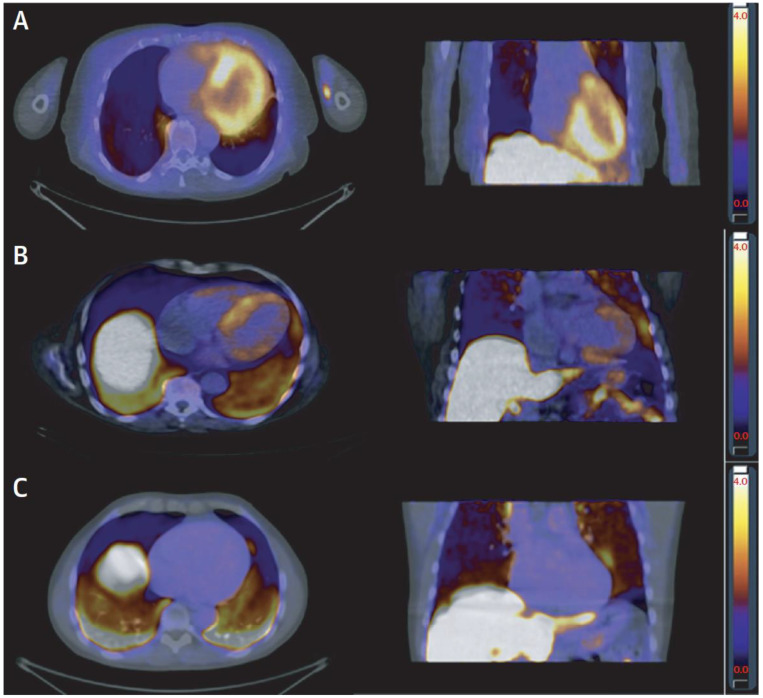
Examples of summed PET images taken 10–20 min after injection of ^11^C-PIB (see below for abbreviations). The signal represents myocardium/blood SUV ratio, and the color scale ranges from black (SUV ratio 0) to white (SUV ratio 4.0). Transaxial and coronal view of (**A**) a patient with AL amyloidosis and high degree of ^11^C-PIB uptake in the left ventricle wall and septum; (**B**) a patient with ATTR amyloidosis and moderate degree of ^11^C-PIB uptake in the cardiac walls, most apparent in the septum; and (**C**) a healthy volunteer with no myocardial ^11^C-PIB uptake. All subjects show high signal in the liver, which is due to normal metabolization of ^11^C-PIB, and in the lungs, which is most likely caused by tracer lipophilicity. ATTR, transthyretin amyloidosis; PET, positron emission tomography; PIB, Pittsburgh compound B; SUV, standardized uptake value. Reproduced, with permission, from [50].

Nuclear imaging with non-perfusion tracers plays an important role in the diagnosis of several less frequent diseases causing heart failure. One of them, which is now treatable, is cardiac amyloidosis. ^99m^Tc-3,3-diphosphono-1,2-propanodicarboxylic acid (DPD)-SPECT (or planar imaging), originally introduced as a “bone scan” for bone imaging, especially bone metastases, is an excellent tool for diagnosing ATTR amyloidosis. However, other forms of amyloidosis, especially AL amyloidosis, are not well detected by DPD imaging, and need blood or urine chemistry or specialized PET tracers [50] for diagnosis (Figure 6). Sarcoidosis, a rare but treatable cause of heart failure, is also well detected by PET, which has higher specificity than CMR for this disease.

## 9. Cardiac Computed Tomography (CT)

The most powerful new imaging modality to emerge in the field of coronary artery disease is cardiac CT. Given a near perfect negative predictive value, it can exclude coronary disease with high reliability. The quantification of the severity of coronary stenoses is more difficult and fraught with the overestimation of the degree of stenosis, and further hampered by previous stent implantation. Artificial intelligence tools such as the available fractional flow reserve (CT-FFR) packages allow a rough non-invasive assessment of fractional flow reserve, but their clinical value is still under evaluation [51]. Apart from the diagnosis/exclusion of coronary artery disease, there is presently little role for CT in the routine workup of heart failure [52].

## 10. Pulmonary Hypertension

The evaluation of right ventricular systolic pressure is part of the standard echocardiographic examination, and, thus, the first step in the diagnostic work-up of suspected pulmonary hypertension. In the presence of pulmonary hypertension, echocardiography also allows to rule in or rule out left-sided diseases, such as valvular disease or LV systolic or diastolic dysfunction, as possible etiologies of pulmonary hypertension, although sometimes echocardiographic data are not conclusive, and invasive diagnostics by right-heart catheterization with exercise are necessary [53]. Furthermore, there is an overlap between classic primary pulmonary hypertension with normal pulmonary capillary wedge pressure and increased pulmonary vascular resistance on the one side and classic secondary pulmonary hypertension with increased wedge pressure and normal vascular resistance. Some patients show features of both categories, a condition termed “combined pre- and post-capillary pulmonary hypertension” [54]. Such “overlap” patients cannot be diagnosed with certainty using echocardiography alone, and need invasive workup.

## 11. How Should Cardiac Imaging Be Used “Wisely” in the Work-Up of Heart Failure?

Echocardiography remains the cornerstone of imaging in heart failure, and is considered mandatory by guidelines as an initial test. Apart from furnishing global functional measures of systolic and diastolic LV, as well as RV, function, echocardiography, in the majority of cases, detects probable underlying causes for heart failure, e.g., myocardial hypertrophy, infarct scars, valvular heart disease, and others. Nevertheless, echocardiography is often just the initial diagnostic step (see Table 1). Detailed information about myocardial structure and perfusion requires additional imaging tailored to the specific abnormalities and clinical circumstances. In this context, CMR allows (a limited) tissue characterization, and is strongly recommended by current heart failure guidelines “in selected cases”, in particular with regard to the presence of myocarditis, storage diseases, iron overload/hemochromatosis, sarcoidosis, and certain cardiomyopathies such as hypertrophic, non-compaction, arrhythmogenic, and others [2]. Information about global and regional myocardial perfusion, including perfusion reserve, is available from CMR, SPECT, and PET, and may indicate the presence of epicardial coronary artery disease, microvascular disease, or another pathology.

## 12. Summary

Recent developments across all modalities of cardiac imaging have impacted the management of heart failure. The field has moved from just providing global information on LV performance, such as ejection fraction, to a multilayered diagnostic effort providing, in some cases, near-histologic diagnosis, and monitoring treatment success, e.g., the regression of amyloidosis [55]. Hence, it is important that the availability of modalities, as well as the competence in their application and interpretation, are safeguarded by professional and healthcare policies. A cooperative “cardiac imager team”, bridging the gaps between cardiology, radiology, and nuclear medicine, is crucial to support the clinicians, and should be established in larger institutions. On the other hand, we must avoid the overuse of imaging, which is mostly due to a lack of a clear understanding of the strengths and weaknesses of particular modalities. A good example for such a strategy is the American College of Cardiology’s “Choosing wisely” [56] campaign in cardiac imaging, and other bodies and countries should follow their example. Finally, it is important that imaging exams, if indicated, are performed with contemporary technology and interpretation skills in order to extract the maximum amount of useful information from each performed exam.

## Figures and Tables

**Table 1:** Goals, strengths, and limitations of cardiac imaging modalities in heart failure.

Modality	Goals	Strengths	Weaknesses/Limitations
**Echocardiography**	Measurement of systolic and diastolic function parameters (EF, GLS, etc.), identification of etiology (e.g., cardiomyopathies, coronary artery disease, hypertrophy, valvular heart disease)	Ubiquitous and prompt availability (first-line imaging modality for heart failure), good assessment of valvular heart disease, cost-effective, no side effects	High observer and test–retest variability, no tissue characterization, only indirect assessment of coronary artery disease by stress test, often limited by image quality
**Cardiovascular Magnetic Resonance**	Measurement of systolic and diastolic function parameters (LV and RV volumes, EF, GLS, etc.), identification of etiology of heart failure (ischemic scar, cardiomyopathy, myocarditis, storage disease, sarcoidosis, and others)	Tissue characterization by late enhancement and tissue relaxation parameters (T1, T2, T*); high volumetric accuracy (gold standard); ability to reliably quantify valvular regurgitation	Not everywhere available, expensive, limited in atrial fibrillation, some contraindications (renal failure for contrast application, some implanted devices, claustrophobia)
**Cardiac Computed Tomography**	Assessment of coronary artery disease; second-line imaging of structural disease (cardiomyopathies, valve disease)	Very high sensitivity for coronary artery disease; very high morphologic resolution	Radiation exposure, renal failure contraindication for contrast application
**Nuclear Perfusion Imaging**	Assessment of myocardial perfusion (coronary or microvascular disease); assessment of amyloidosis with specific tracers; sarcoidosis	PET is gold standard for myocardial perfusion; identification of perfusion/metabolism mismatch, as in hibernating myocardium; high accuracy for amyloidosis and sarcoidosis; DPD imaging (planar/SPECT) is sensitive for cardiac ATTR amyloidosis	PET limited to centers with cyclotron; radiation exposure
